# A New Benzo[6,7]oxepino[3,2-b] Pyridine Derivative Induces Apoptosis in Canine Mammary Cancer Cell Lines

**DOI:** 10.3390/ani14030386

**Published:** 2024-01-25

**Authors:** Natamon Jianpraphat, Wachiraphan Supsavhad, Paiboon Ngernmeesri, Kannika Siripattarapravat, Sirikul Soontararak, Nattaphong Akrimajirachoote, Napasorn Phaochoosak, Usuma Jermnak

**Affiliations:** 1 Department of Pharmacology, Faculty of Veterinary Medicine, Kasetsart University, Bangkok 10900, Thailand; natamon.ji@ku.th (N.J.); fvetanp@ku.ac.th (N.P.); 2 Department of Pathology, Faculty of Veterinary Medicine, Kasetsart University, Bangkok 10900, Thailand; wachiraphan.s@ku.th (W.S.); fvetkks@ku.ac.th (K.S.); 3 Department of Chemistry, Faculty of Science, Kasetsart University, Bangkok 10900, Thailand; paiboon.n@ku.th; 4 Department of Companion Animal Clinical Sciences, Faculty of Veterinary Medicine, Kasetsart University, Bangkok 10900, Thailand; sirikul.s@ku.th; 5 Department of Physiology, Faculty of Veterinary Medicine, Kasetsart University, Bangkok 10900, Thailand; fvetnpa@ku.ac.th

**Keywords:** apoptosis, benzo[6,7]oxepino[3,2-b] pyridine, canine mammary cancer, derivative, induce

## Abstract

**Simple Summary:**

Canine mammary cancer (CMC) is the most prevalent neoplasm in intact female dogs, with approximately 50% of these cases diagnosed as malignant. Various chemotherapeutic agents have been applied in CMC treatment, but these agents come with notable disadvantages and limited information on efficacy. The benzo[6,7]oxepino[3,2-b] pyridine (BZOP) derivative, 2-((2-methylpyridin-3-yl)oxy)benzaldehyde (MPOBA), exhibited potent anticancer activity against a human colorectal cancer cell line. However, the anticancer effects and molecular mechanisms of MPOBA on CMC have not been elucidated. This study investigated the effects of MPOBA on proliferation, migration, and apoptosis and investigated its potential mechanism in two CMC cell lines, REM134 canine mammary carcinoma and CMGT071020 canine mammary tubulopapillary carcinoma. Furthermore, an in vitro cytotoxicity test was also evaluated in the Madin–Darby canine kidney (MDCK) cell line. The results revealed that MPOBA significantly inhibited proliferation and migration and induced apoptosis in CMC cell lines. In addition, MPOBA showed lower cytotoxicity in MDCK cells. According to the gene expression analysis, the mRNA expression levels of TP53 tumor suppressor (*TP53*), Bcl-2 associated X (*BAX*), and B-cell lymphoma-2 (*BCL-2*) in both CMC cells were significantly altered in the treatment groups compared to the control. These results suggested that MPOBA may have induced apoptosis in both CMC cell lines. This effect may be mediated through the downregulation of *BCL-2* and upregulation of *BAX* gene expressions. These findings indicated that MPOBA may serve as an emerging candidate agent for CMC treatment. However, mRNA expression levels may not be directly proportional to protein expression levels. Therefore, additional studies, including apoptosis-related protein analysis and apoptosis marker assays, are required to confirm the major apoptosis signaling pathway regulated by MPOBA.

**Abstract:**

CMC is the most frequently diagnosed cancer and one of the leading causes of death in non-spayed female dogs. Exploring novel therapeutic agents is necessary to increase the survival rate of dogs with CMC. MPOBA is a BZOP derivative that has a significant anticancer effect in a human cell line. The main goal of this study was to investigate the anticancer properties of MPOBA against two CMC cell lines (REM134 and CMGT071020) using a 3-(4,5-Dimethyl-2-thiazolyl)-2,5-diphenyl-2H-tetrazolium bromide (MTT) assay, a wound healing assay, a transwell migration assay, an Annexin V-FITC apoptosis assay with a flow cytometry analysis, a mRNA expression analysis using quantitative real-time PCR (qRT-PCR), and an immunohistochemistry (IHC). According to the accumulated studies, MPOBA caused significant concentration- and time-dependent reductions in cell proliferation and cell migration and induced apoptosis in both CMC cell lines. In gene expression analysis, nine canine genes, including *TP53*, *BCL-2*, *BAX*, epidermal growth factor receptor (*EGFR*), snail transcription factor (*SNAIL*), snail-related zinc-finger transcription factor (*SLUG*), *TWIST*, *E-cadherin*, and *N-cadherin*, were investigated. The mRNA expression results revealed that MPOBA induced upregulation of *TP53* and overexpression of the pro-apoptotic gene *BAX*, together with an inhibition of *BCL-2.* Moreover, MPOBA also suppressed the mRNA expression levels of *SNAIL*, *EGFR*, and *N-cadherin* and induced upregulation of *E-cadherin*, crucial genes related to the epithelial-to-mesenchymal transition (EMT). However, there was no significant difference in the IHC results of the expression patterns of vimentin (VT) and cytokeratin (CK) between MPOBA-treated and control CMC cells. In conclusion, the results of the present study suggested that MPOBA exhibited significant anticancer activity by inducing apoptosis in both CMCs via upregulation of *TP53* and *BAX* and downregulation of *BCL-2* relative mRNA expression. MPOBA may prove to be a potential candidate drug to be further investigated as a therapeutic agent for CMC.

## 1. Introduction

Longer lifespans in both humans and dogs are associated with an increased risk of cancer [1]. Canine mammary tumors (CMTs) are the most common tumors in intact female dogs. Among CMTs, approximately 50% of these neoplasms are diagnosed as malignant [2]. Clinical and molecular similarities between human breast cancer (HBC) and canine mammary cancer (CMC) have been reported [3]. There are multiple risk factors for the development of these cancers, such as hormonal exposure, age, genetics, and nutritional factors [4]. Furthermore, the development and progression of these cancers are linked to several crucial genes that govern processes such as cell proliferation, migration, and invasion, as well as apoptosis [5]. In CMC, changes in the expression levels of numerous genes, including *TP53*, *BAX*, *BCL-2*, *SNAIL*, *SLUG*, *TWIST*, *E-cadherin*, *N-cadherin*, and *EGFR*, have been reported [6,7,8]. One of the core regulators in the early phase of apoptosis induction is *TP53*. Several genes involved in the downstream signaling of *TP53* and other triggers of apoptosis have been demonstrated. The upregulation of *TP53* can induce apoptosis in CMC [9]. A positive correlation between the evasion of apoptosis and *BCL-2* expression was previously observed in CMC, suggesting that the BCL-2 protein prevents apoptosis through BAX inhibition [10]. The EMT has been recognized as a significant contributor to tumor metastasis and progression. In the process of EMT, epithelial cells undergo a series of biochemical changes to acquire more migratory mesenchymal-cell-like properties [11]. This transformation is marked by enhanced migratory capacity, invasiveness, and an increased production of extracellular matrix components [12]. During EMT, several mechanisms have been described, including the association between *E-cadherin* downregulation and *N-cadherin* upregulation and tumor development as well as progression. Classical EMT transcription factors, including SNAIL, SLUG, and TWIST, can induce EMT to promote cancer invasion and metastasis [13]. In addition, EGFR activation is also related to aggressiveness in malignant CMC, including increased angiogenesis, large tumor sizes, tumor necrosis, higher mitotic rates, advanced clinical stages, and malignancy [14].

Currently, the practical treatment for CMC is surgical removal, consisting of removing the tumor and its regional lymph nodes to prevent potential growth and metastasis [15]. Nonetheless, the use of chemotherapy has been described for the management of CMC in cases where these malignant tumors have already metastasized to distant sites [16]. Chemotherapy is usually an important palliative therapy following the surgical treatment of CMC that helps to enhance the overall survival rate and prevent the risk of developing tumor recurrence [17]. Although chemotherapy is a practical treatment to increase the median survival time, it has highly adverse effects, and there is limited information on its efficacy and impacts on resistance [18]. Thus, the effectiveness of treatments for cancer varies significantly depending on factors such as the stage of cancer progression, genetic predisposition, and chemotherapeutic tolerance [19]. The development of new chemotherapeutic agents has contributed to reducing the risk of cancer recurrence and improving animal patient survival.

**Figure 1:**
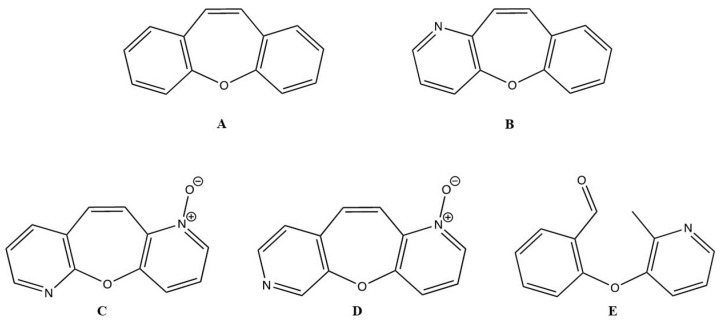
Structures of dibenzo[*b,f*]oxepine (**A**), benzo[6,7]oxepino[3,2-b]pyridine (**B**), oxepino(2,3-b:6,7-b’)dipyridine 1-oxide (**C**), oxepino(3,2-b:7,6-c’)dipyridine 1-oxide (**D**), and 2-((2-methylpyridin-3-yl)oxy)benzaldehyde (**E**).

Dibenzo[*b*,*f*]oxepine (DO) (Figure 1A) is an important structure in natural and medicinal chemistry, and its derivatives are found in various medicinally relevant plants [20,21]. Several compounds with DO rings have been reported to exhibit potent biological activities, including analgesic properties [22], antioxidant properties [23], antimycobacterial activity [24], anti-inflammatory activity [25], antidepressant activity [26], anti-apoptotic properties [27], and anticancer activities [28,29]. Studies of the pharmacological properties of DO derivatives have received much attention based on their fascinating structural features and health-promoting functions. For example, bauhinoxepins A and B isolated from the roots of *Bauhinia saccocalyx* exhibited significant growth inhibition against several human cancer cell lines [30]. Pacharin and Bauhiniastatin-1 isolated from *Bauhinia acuruana* induced apoptosis in HBC cells [31]. Various anticancer mechanisms of action of DO derivatives have been published for the past few years. The synthesized DO derivative, 1-(2-(4-(dibenzo[b,f]thiepin-10-yl)phenoxy)ethyl)piperidine (DTPEP), which mimics the structure of tamoxifen, exhibited greater potency in inhibiting the growth of human mammary adenocarcinoma cell lines (MCF-7 and MDA-MB-231). DTPEP increased the expression of levels of cytochrome c and BAX and decreased BCL-2 proteins in both HBCs. It induced both early and late apoptosis in HBCs through ROS-dependent apoptosis through both the extrinsic and intrinsic pathways [32] and downregulation of PI3K/Akt and PKCα expression [33]. Furthermore, the analog structures of DO derivatives with benzene rings were found to be contributing to inhibiting microtubules in HBC cell lines (MDA-MB0-468, MCF-7, and HS578T) with a less than half-maximal inhibitory concentration (IC_50_) values [34]. Because of the importance of the DO core as a pharmacophore, wide synthetic efforts have been made [35]. Benzo[6,7]oxepino[3,2-b]pyridine (BZOP) (Figure 1B) is an analog of DO. Researchers became interested in its synthesis because it is easily prepared in a one-step reaction that provides moderate-to-good yields, making the compound ideal for large-scale production. Pyridine derivatives have garnered significant interest in contemporary medical research due to their potential for treating diversified serious diseases, including breast cancer, myeloid leukemia, and idiopathic pulmonary fibrosis [36,37]. These functional groups exhibit cytotoxic and anti-proliferative properties against various cancer cells through the activation of the apoptosis pathway, as demonstrated in studies involving 2-methylpyridine-1-ium-1-sulfonate, pyrano[3,2-c]pyridines, and their derivatives. These compounds have been identified as potent suppressors of HBC cell lines and robust inducers of apoptosis [38,39]. Moreover, some BZOP derivatives were reported to exhibit anticancer activity against a human colorectal cancer cell line (HCT-116) with a low IC_50_ value [40]. However, there is limited information on the anticancer potential of BZOP derivatives in CMC cell lines. In addition, the molecular mechanism of action that may contribute to their anticarcinogenic effects has not yet been elucidated.

In this study, the cytotoxic effects of three BZOP derivatives—oxepino(2,3-b:6,7-b’)dipyridine 1-oxide (OPDOb) (Figure 1C), oxepino(3,2-b:7,6-c’)dipyridine 1-oxide (OPDOc) (Figure 1D), and 2-((2-methylpyridin-3-yl)oxy)benzaldehyde (MPOBA) (Figure 1E)—were screened using two CMC cell lines (REM134 and CMGT071020) and one normal dog kidney cell line (MDCK). Furthermore, the inhibition of migration and the induction of apoptosis, as well as the molecular mode of action of the most active BZOP derivative, MPOBA, were investigated in two CMC cell lines.

## 2. Materials and Methods 

### 2.1. Synthesis of BZOP Derivatives

The synthesis and characterization of three BZOP derivatives, including OPDOb, OPDOc, and MPOBA, were described in detail by Thongaram et al. [40].

### 2.2. Cell Lines and Cell Cultures

REM134 canine mammary carcinoma, CMGT071020 canine mammary tubulopapillary carcinoma, and MDCK Madin–Darby canine kidney cell lines were used in this study. REM134 cells were obtained from the ECACC (Cat#12122002, UK), and MDCK cells were obtained from the ATCC (CRL-2936, Manassas, VA, USA), while CMGT071020 cells were previously isolated from a mammary tumor of a 7-year-old female Siberian husky at Kasetsart University Animal Cell Bank, Faculty of Veterinary Medicine, Kasetsart University. All cell lines were grown in Dulbecco’s modified Eagle’s medium (DMEM) (Corning, New York, NY, USA) supplemented with 4.5 g/L glucose and L-glutamine; 10% fetal bovine serum (FBS) (Gibco, New York, NY, USA) and a 1% antibiotic–antimycotic solution (Gibco, New York, NY, USA) composed of the antibiotics penicillin (10,000 IU/mL), streptomycin (10,000 μg/mL), and amphotericin B (2.5 µg/mL) in an atmosphere of 5% CO_2_ with 95% humidity at 37 °C. After reaching 80% confluence, cells were harvested with 0.25% TrypLE (Gibco, New York, NY, USA) and sub-cultured. The mycoplasma statuses in the cell lines were investigated using a MycoSensor QPCR assay kit (Agilent, Santa Clara, CA, USA) according to the manufacturer’s protocols. All cell lines used in this study were free from mycoplasma contamination.

### 2.3. Cell Proliferation Test

The cytotoxic effects of the three BZOP derivatives were studied via the 3-(4,5-Dimethyl-2-thiazolyl)-2,5-diphenyl-2H-tetrazolium bromide (MTT) assay. The REM134, CMGT071020, and MDCK cell lines were seeded at a density of 5 × 10^3^ cells/well in 96-well plates and allowed to grow for 24 h. They were then treated with the three BZOP derivatives at various concentrations (0–300 µM) for 24 or 48 h, whereas cells treated with the culture medium containing 0.1% dimethyl sulfoxide (DMSO) were used as a negative control as the compounds were dissolved in DMSO. Subsequently, the cells were incubated with 10 µL of an MTT solution (Sigma, St. Louis, MO, USA) for 4 h. After the incubation with MTT solution, 100 µL of a solubilization solution was added to dissolve water-insoluble purple formazan crystals, and the plate was incubated overnight at 37 °C in 5% CO_2_. The absorbance of the formazan solution was measured using a Synergy H1 hybrid multi-mode microplate reader (BioTek, Winooski, VT, USA) at 570 nm. The experiment was conducted with four replicates, and the relative cell viability (%) of the treated cells was expressed as a percentage relative to the untreated control cells, which were considered to have 100% viability.

### 2.4. Morphological Analysis of REM134 and CMGT071020 Cell Lines

To investigate the morphological changes of the two CMC cell lines during the treatment with MPOBA, they were cultured in a 6-well plate at a density of 2 × 10^5^ cells/well and allowed to incubate for 24 h until they reached 70–80% confluence. They were subsequently treated with MPOBA at varying concentrations from 0 to 200 µM for 48 h. Images of the cells were captured using a phase-contrast inverted microscope (Nikon, Tokyo, Japan) at 40× magnification.

### 2.5. Cell Migration Assay

The effect of MPOBA on cell migration was examined using a two-dimensional wound healing assay. The REM134 and CMGT071020 cell lines were seeded in a 6-well plate at a density of 5 × 10^5^ cells/well and allowed to incubate for 24 h until they reached 100% confluence. The cells were scratched using a sterile 1 mL pipette tip and then treated with MPOBA at concentrations ranging from 0 to 200 µM for 24 h. Images of the scratches were captured at 0, 12, and 24 h post-treatment using phase-contrast microscopy. The wound areas were checked and measured using the NIS-Elements D imaging software program version 5.2 (Nikon, Tokyo, Japan). The cell migration ability was calculated as a percentage of the area covered by cells at 24 h compared to the initial wound area at 0 h. The experiments were repeated four times.

### 2.6. Transwell Migration Assay

The CMC cell migration assay was carried out using Transwell 24-well microplates with an 8 µm pore size (Costar, Corning, NY, USA). Both CMC cell lines were seeded into the upper Transwell insert at a density of 5 × 10^3^ cells/well, containing various concentrations of MPOBA (0–200 µM) with 5% FBS in DMEM media. Then, 10% FBS in DMEM medium was added into the lower chambers, and the cells were incubated for 24 h. After an incubation duration of 24 h, the non-invaded cells were removed with a cotton swab from the upper surface of the membrane, and the invaded cells on the lower surface of the membrane were fixed with 100% methanol for 1 min and stained with the Wright–Giemsa stain for 20 min. The number of migrated cells was averaged by counting five random microscope fields for each sample at 4 × magnification in four independent experiments.

### 2.7. Apoptosis Assay 

The mode of cell death induced by MPOBA was evaluated using FITC Annexin V apoptosis detection kit I (BD Bioscience, San Jose, CA, USA) according to the manufacturer’s protocols. The REM134 and CMGT071020 cell lines were seeded at a density of 2 × 10^4^ cells/well in a 12-well plate and allowed to grow for 24 h (60% confluency) prior to treatment with MPOBA at concentrations ranging from 0 to 200 µM for 48 h. After the incubation period, all adhering and floating cells were harvested and transferred into sterile centrifuge tubes. The cells were centrifuged at 400× *g* for 5 min at room temperature. The supernatant was aspirated, leaving approximately 50 µL of residual fluid in the tubes to avoid disturbing the pellets. FITC Annexin V (5 µL) and PI (5 µL) were added to the tubes containing cell pellets. The mixtures were gently vortexed and incubated for 15 min at room temperature in the dark. A binding buffer (400 µL) was added to each tube. A total of 20,000 events were acquired for each sample using a CytoFLEX flow cytometer (Beckman Coulter, Miami, FL, USA) within an hour. The viable cells exhibited Annexin V-FITC(−)/PI(−); early apoptotic cells exhibited Annexin V-FITC(+)/PI(−); late apoptotic cells exhibited Annexin V-FITC(+)/PI(+); and necrotic cells exhibited Annexin V-FITC (−)/PI (+). The experiment was performed with four replicates. The positive control was doxorubicin-treated CMC cells, while the negative control was 0.1% DMSO-treated CMC cells [41].

### 2.8. RNA Isolation and Quantitative Real-Time PCR (qRT-PCR)

After the treatment of the cells with MPOBA at varying concentrations from 0 to 200 µM for 48 h, the total RNA was isolated using a GeneJet RNA Purification kit (Thermo Fisher Scientific, Waltham, MA, USA) according to the manufacturer’s protocol for adherent tissue culture cells using the spin-column method. The RNA’s concentration and purity (A260/A280 ratio) were spectrophotometrically determined using a NanoDrop One/OneC Microvolume UV-Vis Spectrophotometer (Thermo Fisher Scientific, Waltham, MA, USA). To remove genomic DNA contamination, the samples were treated with RQ1 RNase-Free DNase (Promega, Madison, WI, USA) according to the manufacturer’s instructions. First-strand cDNA was obtained using a SuperScript^®^ III First-Strand Synthesis kit for reverse-transcription polymerase chain reaction (RT-PCR) (Invitrogen, Carlsbad, CA, USA) according to the manufacturer’s protocol. Briefly, total RNA (1 μg), oligo (dT) primers (50 µM), a dNTP mix (10 mM), the cDNA synthesis mix, and nuclease-free water were added to obtain a final volume of 20 µL. PCR amplification was performed using a G-Storm GS482 PCR thermal cycler (Gene Technologies, Somerset, UK) with the following reaction profile: 65 °C for 5 min, 50 °C for 50 min, 85 °C for 5 min, and 37 °C for 5 min. The relative mRNA expression levels of canine *SNAIL*, *SLUG*, *TWIST*, *E*-*cadherin*, *N*-*cadherin*, *TP53*, *EGFR*, *BAX*, and *BCL-2* were normalized to canine glyceraldehyde 3-phosphate dehydrogenase (*GAPDH*). All primers used for qRT-PCR are shown in Table 1. The relative mRNA expression levels were calculated using the 2^–∆∆^ Cq method. The transcriptional levels of all genes were determined in four replicates via qRT-PCR using a CFX 96 Touch real-time PCR detection system (Bio-Rad, Hercules, CA, USA). The qRT-PCR was performed using a reaction mixture containing 2 μL of the cDNA template, 10 μL of the iTaq Universal SYBR Green Supermix (Bio-Rad, Hercules, CA, USA), and 1 μL of each primer. The qRT-PCR protocol for the *SNAIL*, *SLUG*, *TWIST*, *EGFR*, *TP53*, *BAX*, *BCL-2*, and *GAPDH* genes was carried out as follows: initial denaturation at 94 °C for 3 min, denaturation at 94 °C for 15 s, annealing at 60 °C for 30 s, and extension at 72 °C for 1 min (40 cycles), and dissociation curves were generated between 65 °C and 95 °C to assess the specificity of the amplicons [42,43,44]. The qRT-PCR protocol for *E-cadherin* and *N-cadherin* was carried out according to Yu et al. [45].

### 2.9. Immunohistochemistry (IHC)

The cells were cultured in a T-25 cell culture flask (SPL life science, Gyeonggi-do, Republic of Korea) at a density of 2 × 10^5^ cells/well and allowed to incubate for 24 h until they reached 60–70% confluence. They were subsequently treated with MPOBA at varying concentrations from 0 to 200 µM for 48 h. After treatment, cells were harvested using TrypLE and subjected to centrifugation at 1200 rpm for 5 min to obtain cell pellets. Then, the supernatant was replaced with 10% formalin, and the cell pellets were fixed overnight. The fixed cell pellets were embedded in paraffin blocks and sectioned with a thickness of 3 μm. These slide sections were then subjected to the deparaffinization and rehydration processes. For antigen retrieval, a heat-induced method was adopted, utilizing a vegetable steamer in a citrate buffer (pH = 6) at 90–100 °C for 45 min, followed by a cooling period of 30 min. After these preparatory steps, slides were mounted on cover plates within a Sequenza slide rack (Thermo Scientific, Waltham, MA, USA), void of air bubbles, and filled with Tris-buffered saline (TBS) (pH = 7.6). The immunohistochemical detection employed a Novolink™ Polymer Detection Kit (Leica Biosystems, Newcastle, UK) according to the manufacturer’s instructions. This began with the inhibition of endogenous peroxidase activity with a 150 μL peroxidase block at room temperature for 5 min, followed by washing with TBS three times for 3 min each. Subsequently, a 150 μL protein block was applied, incubated for 5 min, and washed three times with TBS for 3 min. Primary antibodies, including a vimentin (VT) mouse anti-human monoclonal antibody (Clone V9, ready-to-use kit, Leica Biosystems, Newcastle, UK) and a multi-cytokeratin (CK) mouse antibody (Clone AE1/AE3, ready-to-use kit, Leica Biosystems, Newcastle, UK), were incubated at 37 °C for 1 h. Then, the slides were incubated with 150 μL of a post-primary solution, a rabbit anti-mouse secondary antibody, at room temperature for 15 min, followed by 15 min of incubation with 150 μL of anti-rabbit Poly- horseradish peroxidase (HRP). After that, the slides were developed with diaminobenzidine (DAB) at room temperature for 10 s, followed by counterstaining with hematoxylin for 3 min. The dehydrated and mounted slides were subsequently subjected to imaging using an Olympus BX61VS slide scanner (Olympus Optical, Tokyo, Japan) at 40× magnification.

### 2.10. Analysis and Interpretation of IHC 

A quantitative evaluation of the stained slides was performed utilizing the Allred scoring method [46,47] and QuPath bioimaging software version 0.4.4 [48] to mitigate potential biases. The image type of brightfield (H-DAB) was selected with 40× magnification and 0.17 µm pixels. The hematoxylin stain and DAB stain were annotated using the rectangle tool and the grid tool to randomly select 10 fields with dimensions of 25 μm × 25 μm. The optimal stain separation in the brightfield images was obtained using the “estimate stain vectors” command in the analyze menu (Figure S1). The positive cells were systematically detected using the “positive cell detection” command in the analyze menu. Multiple threshold parameters were set, as shown in Figure S2. The Allred score was calculated according to the quantity of marked cells and the intensity of the staining, as shown in Table S1. The scores ranged between 0 and 8. Samples with scores of <2 were considered negative, and samples with scores of 2–8 were considered positive. The scores and criteria used to quantify the labeled cells were 0, no labeling; 1, labeling in <1% of cells; 2, labeling in 1–10% of cells; 3, labeling in 11–33% of cells; 4, labeling in 34–66% of cells; and 5, labeling in 67–100% of cells. The criteria and scores used to determine the intensity of the immunostaining were negative, 0; weak, 1; moderate, 2; and high, 3. The staining intensity was determined visually. The sum of these criteria for each sample determined the final score.

### 2.11. Statistical Analysis 

The data were expressed as the mean ± SD from four independent experiments and were analyzed using GraphPad Prism 6.0 (GraphPad Software, Inc.; La Jolla, CA, USA). Statistical differences between groups were determined using the Kruskal–Wallis test, followed by Dunn’s post hoc test for multiple comparisons. A *p*-value < 0.05 (*p* < 0.05) was considered to indicate a statistically significant difference.

## 3. Results

### 3.1. Antiproliferation Effects of BZOP Derivatives on Three Cell Lines

**Figure 2:**
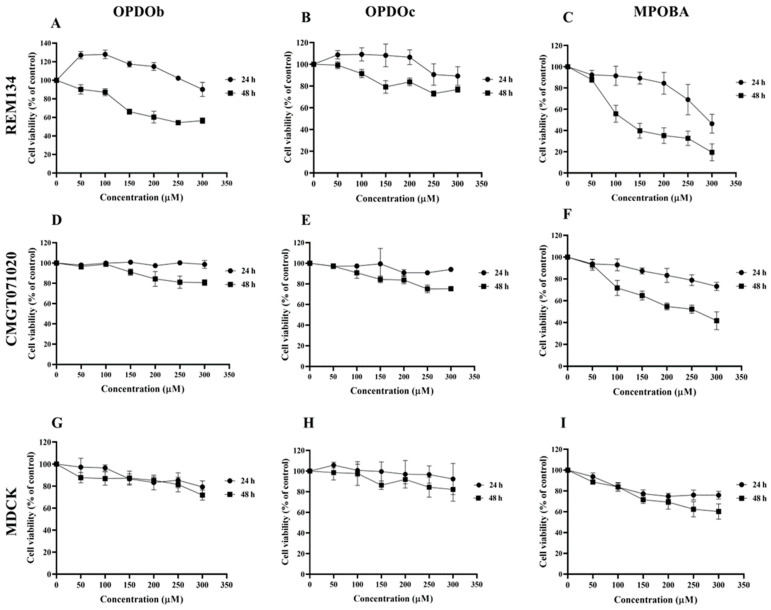
The cytotoxic effects of oxepino(2,3-b:6,7-b’)dipyridine 1-oxide (OPDOb), oxepino(3,2-b:7,6-c’)dipyridine 1-oxide (OPDOc), and 2-((2-methylpyridin-3-yl)oxy)benzaldehyde (MPOBA) on two CMC cell lines (REM134 (**A**–**C**) and CMGT071020 (**D**–**F**) cells) and a normal canine kidney MDCK cell line (**G**–**I**) based on MTT assay after 24 or 48 h of treatment. Each data point indicates the mean of four separate experiments ± SD.

The effects of three BZOP derivatives on two CMC cell lines (REM134 and CMGT071020) and a normal canine kidney (MDCK) cell line were studied via MTT assay. In this experiment, all cells were treated with seven serial concentrations (0 –300 μM) of compounds (OPDOb, OPDOc, and MPOBA) for 24 or 48 h (Figure 2). The IC_50_ values of three BZOP derivatives at 24 and 48 h in REM134, CMGT071020, and MDCK cells are shown in Table 2. There were no significant differences in the IC_50_ profile pattern in all cell lines at 24 h of treatment. According to the IC_50_ values at 48 h, MPOBA showed the strongest cytotoxic effect on REM134 cells (IC_50_ value of 87 µM) and CMGT071020 cells (IC_50_ value of 104 µM), followed by OPDOb and OPDOc, respectively. In addition, MPOBA showed lower cytotoxicity in MDCK cells (IC_50_ value of 267 µM) than in the CMC cell lines. This result clearly demonstrated that MPOBA caused a significant concentration- and time-dependent reduction in the cell proliferation of both CMC cell lines.

### 3.2. Effect of MPOBA on Morphology of Both CMC Cell Lines

**Figure 3:**
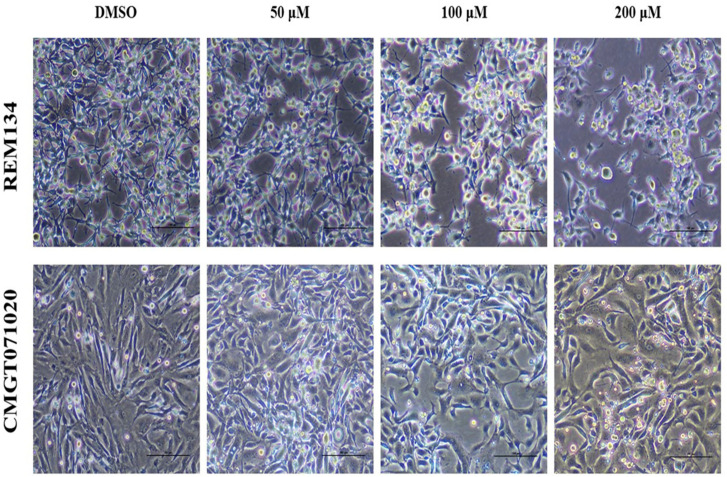
Morphological changes in MPOBA-treated REM134 and CMGT071020 cells after 48 h of treatment with various concentrations of MPOBA. Decrease in cell population, cell shrinkage, rounding, vacuolation, and a loss of cell-to-cell adhesion were noted with the increase in the concentration of MPOBA. Cells were observed using phase-contrast inverted microscope at 40× magnification (scale bar 100 µm).

In both CMC cell lines, applying MPOBA at 50, 100 or 200 µM for 48 h resulted in reduced in cell size, cell shrinkage, rounding, vacuolation, and a loss of cell-to-cell adhesion and some cells were detached from the culture plate (Figure 3), while the cell morphologies in control (0.1% DMSO) showed no difference.

### 3.3. Anti-Migration Effect of MPOBA on Both CMC Cell Lines

**Figure 4:**
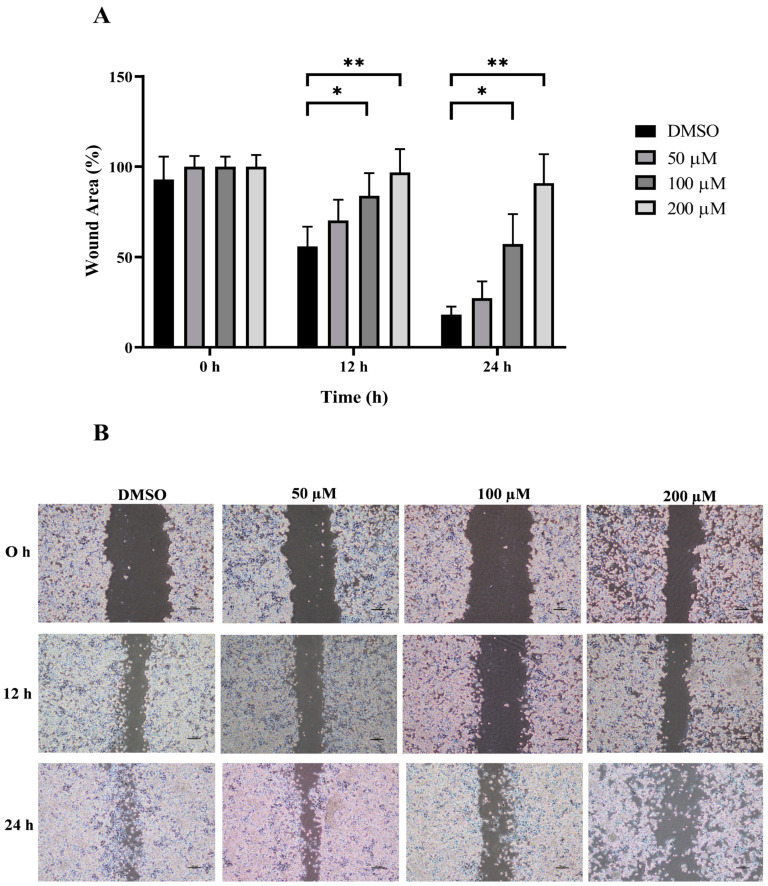
Effect of MPOBA treatment on the migration ability of REM134 cells. Cells were treated with MPOBA at concentrations ranging from 0 to 200 µM for 0, 12, or 24 h. The percentage of wound closure area was determined by comparing the change in wound closure area to that of the untreated control at 0 h (**A**). The data were expressed as the mean ± SD values of four independent experiments. Differences among groups were analyzed using a Kruskal–Wallis test followed by Dunn’s multiple comparisons post hoc tests (* *p* < 0.05, ** *p* < 0.01 vs. untreated control). Cell migration was assessed by a wound healing assay using phase-contrast microscopy (**B**).

**Figure 5:**
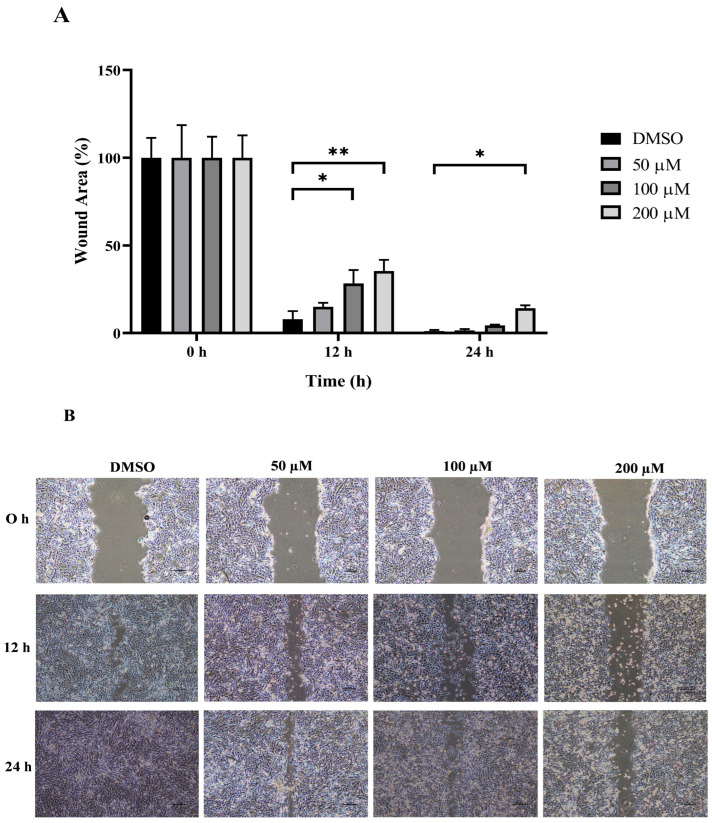
Effect of MPOBA treatment on the migration ability of CMGT071020 cells. Cells were treated with MPOBA at concentrations ranging from 0 to 200 µM for 0, 12, or 24 h. The percentage of wound closure area was determined by comparing the change in wound closure area to that of the untreated control at 0 h (**A**). The data were expressed as the mean ± SD values of four independent experiments. Differences among groups were analyzed using a Kruskal–Wallis test followed by Dunn’s multiple comparisons post hoc tests (* *p* < 0.05; ** *p* < 0.01 vs. untreated control). Cell migration was assessed by a wound healing assay using phase-contrast microscopy (**B**).

**Figure 6:**
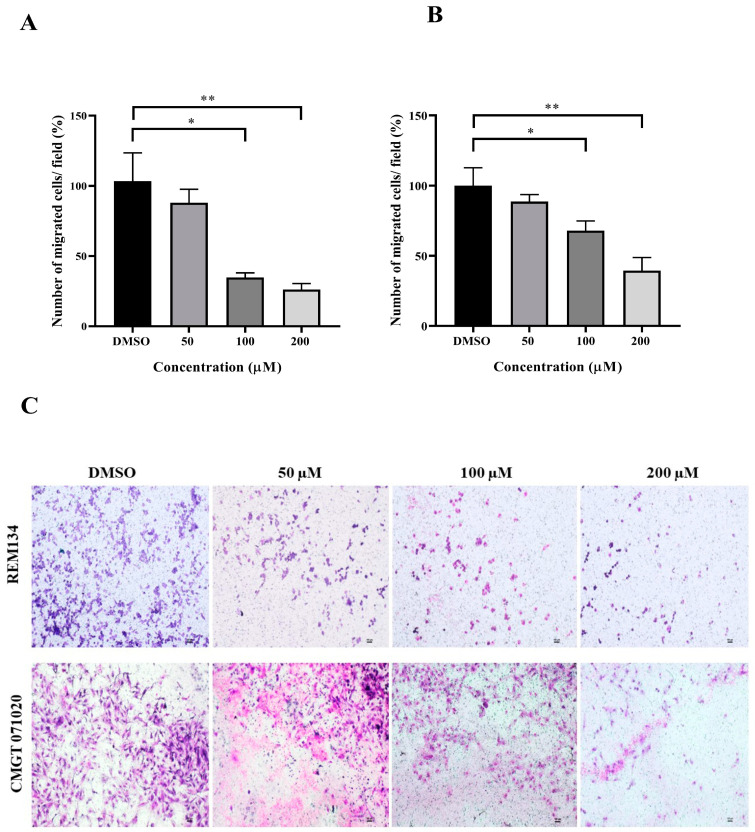
Effects of MPOBA treatment on the migration ability of REM134 and CMGT071020 cells. Cell migration was measured by a transwell migration assay. Cells were treated with various concentrations (0–200 µM) of MPOBA for 24 h. The REM134 and CMGT071020 cell migration percentages were determined by comparing the number of migrated cells after treatment with MPOBA at the various concentrations to that of the control group (0.1% DMSO) (**A**,**B**). The data were expressed as the mean ± SD values of four independent experiments. Differences among groups were analyzed using a Kruskal–Wallis test followed by Dunn’s multiple comparisons post hoc tests (* *p* < 0.05, ** *p* < 0.01 vs. untreated control). Morphology of migrated REM134 and CMGT071020 cells after treatment for 24 h with MPOBA at concentrations of 0–200 µM at 4 × magnification (scale bar 100 µm) (**C**).

The wound healing and transwell assays were carried out to determine whether MPOBA inhibits REM134 and CMGT071020 cell migration. The cell migration distance was measured quantitatively for 24 h as it is directly proportional to the wound closure mechanism. The results of the wound healing assay showed that MPOBA significantly reduced the migratory ability of wound healing assay CMC cells. A significant decline in cell migration was observed when both CMC cells were exposed to 100 or 200 µM of MPOBA after 12 or 24 h of treatment compared to untreated control cells (Figure 4 and Figure 5). Transwell migration experiments revealed that the percentage of migrated cells in MPOBA-treated groups was lower than that in the untreated control group after 24 h of treatment (Figure 6). After treating cells with 100 or 200 µM MPOBA, the percentages of migrated REM134 cells were 35% and 26%, respectively (Figure 6A)., whereas the percentages of migrated CMGT071020 cells were 68% and 40%, respectively (Figure 6B). Based on the results of wound healing and transwell migration experiments, MPOBA could significantly decrease the migration ability of CMC cells in a concentration-dependent manner.

### 3.4. Effect of MPOBA on Apoptotic Induction in REM134 and CMGT071020 Cells 

**Figure 7:**
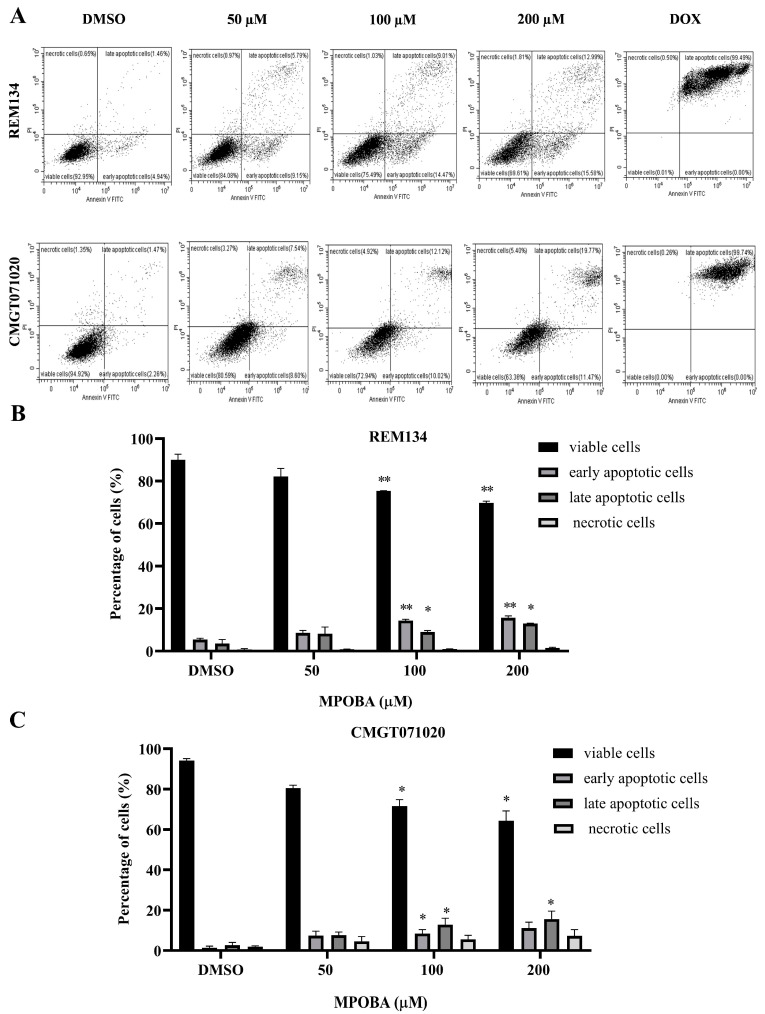
Flow cytometry analysis of REM134 and CMGT071020 cells after 48 h of treatment with various concentrations of MPOBA obtained from a count of 20,000 events (**A**). Percentage of apoptotic MPOBA-treated REM134 cells after 48 h of treatment (**B**). Percentage of apoptotic MPOBA-treated CMGT071020 cells after 48 h of treatment (**C**). The apoptotic REM134 and CMGT071020 cells were calculated as the percentage of apoptotic cells compared with the total number of cells. The data were expressed as the mean ± SD of four independent experiments (* *p* < 0.05, ** *p* < 0.01 vs. untreated control). The positive control was doxorubicin-treated CMC cells (DOX); the negative control was 0.1% DMSO-treated CMC cells (DMSO).

The apoptotic effect of MPOBA was examined by double staining both CMC cell lines with Annexin V-FITC/PI and evaluating them using flow cytometry (Figure 7A). Cells were treated with different concentrations (0–200 µM) of MPOBA for 48 h. The results demonstrated that MPOBA significantly induced apoptotic cell death after 48 h of treatment. The percentage of total apoptosis in REM134 cells increased from 5.14% in the untreated cells to 23.6%, or 28.75%, by adding 100 or 200 µM MPOBA (Figure 7B). Applying MPOBA at the same concentrations also significantly increased the percentage of total apoptosis in CMGT071020 cells from 5.97% in the untreated cells to 21.88% or 27.07%, respectively (Figure 7C). Therefore, our data demonstrated that MPOBA significantly induced cell apoptosis in both CMC cell lines.

### 3.5. Effect of MPOBA on Relative mRNA Expression Levels in Both CMC Cell Lines

**Figure 8:**
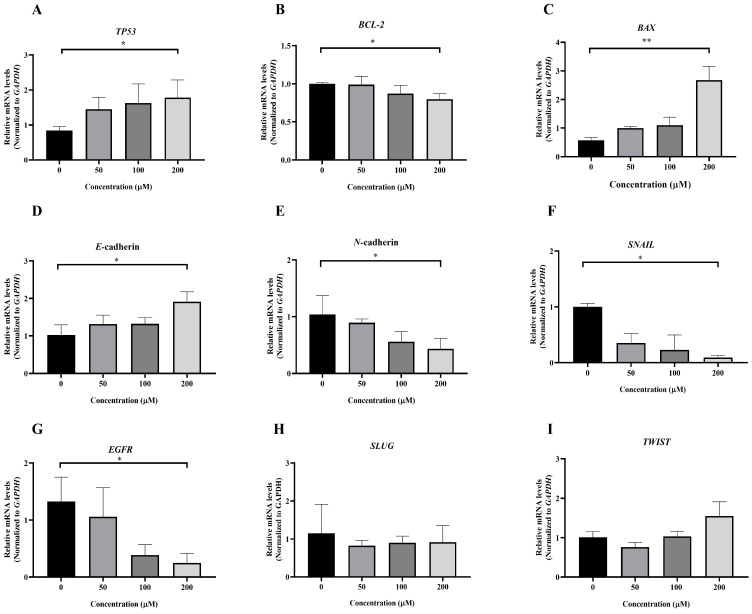
Effects of MPOBA treatment on *TP53*, *BCL-2*, *BAX*, *E-cadherin*, *N-cadherin*, *SNAIL*, *EGFR*, *SLUG*, and *TWIST* mRNA expression in REM134 cells (**A**–**I**). Cells were treated with different concentrations of MPOBA for 48 h. The relative abundances of mRNA were normalized by *GAPDH* mRNA expression. The data were expressed as the mean ± SD values of four independent experiments. Differences among groups were analyzed using a Kruskal–Wallis test followed by Dunn’s multiple comparisons post hoc tests (* *p* < 0.05, ** *p* < 0.01 vs. untreated control).

**Figure 9:**
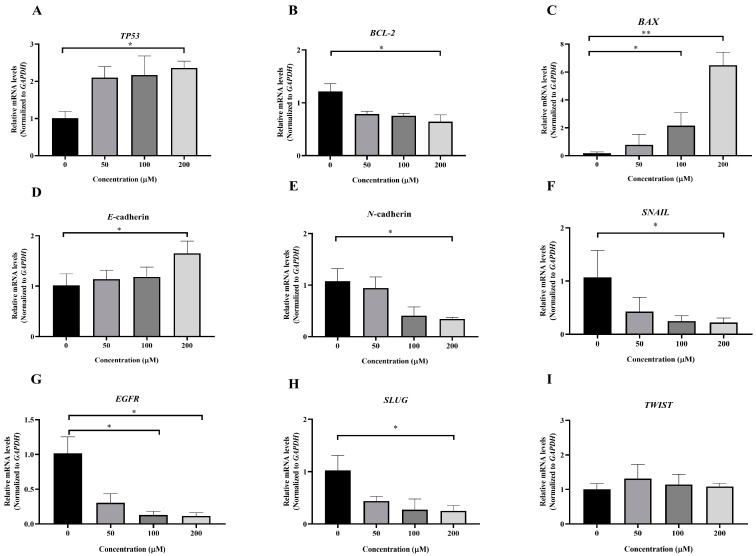
Effects of MPOBA treatment on *TP53*, *BCL-2*, *BAX*, *E-cadherin*, *N-cadherin*, *SNAIL*, *EGFR*, *SLUG*, and *TWIST* mRNA expression in CMGT071020 cells (**A**–**I**). Cells were treated with different concentrations of MPOBA for 48 h. The relative abundances of mRNA were normalized by *GAPDH* mRNA expression. The data were expressed as the mean ± SD values of four independent experiments. Differences among groups were analyzed using a Kruskal–Wallis test followed by Dunn’s multiple comparisons post hoc tests (* *p* < 0.05, ** *p* < 0.01 vs. untreated control).

To investigate the molecular mechanism underlying MPOBA-induced apoptosis in REM134 and CMGT071020 cells, the mRNA expression levels of apoptosis-related genes, including *TP53*, *BCL-2*, and *BAX*, in both CMC cell lines were assessed using qRT-PCR. Figure 8A and Figure 9A illustrate that the expression level of *TP53* mRNA significantly increased in CMC cells treated with MPOBA at a concentration of 200 µM for 48 h compared to the control. In Figure 8B and Figure 9B, it is evident that the mRNA expression level of the *BCL-2* gene significantly decreased, while the mRNA expression level of the *BAX* gene was dramatically increased in cancer cells treated with MPOBA at a concentration of 200 µM (Figure 8C and Figure 9C). These findings suggested that MPOBA primarily induced apoptosis in REM134 and CMGT071020 cells through the regulation of these genes.

The EMT was identified as a complex molecular and cellular process crucial for tissue remodeling. It plays vital roles in numerous biological and pathological processes, including embryonic development, wound healing, cancer cell invasion, migration, and drug resistance in cancer [11,12]. To further verify the inhibitory effect of MPOBA on EMT, the mRNA expression levels of EMT-related genes, including *SNAIL*, *SLUG*, *TWIST*, *E*-cad*herin*, and *N*-cadherin, were assessed in both CMC cell lines. Following MPOBA treatment, there was an observed upregulation of the mRNA expression level of the epithelial marker *E-cadherin* (Figure 8D and Figure 9D), while the mRNA expression levels of the mesenchymal genes *N*-cadherin (Figure 8E and Figure 9E), *SNAIL* (Figure 8F and Figure 9F), and *SLUG* (Figure 9H) exhibited a significant downregulation. Although the mRNA expression level of the *SLUG* gene was not significantly different between the treated and untreated REM134 cells, it presented a decreasing tendency in the treated REM134 cells (Figure 8H). However, the relative mRNA expression levels of the *TWIST* gene between the treated and untreated CMC cells were not significantly different (Figure 8I and Figure 9I). These findings indicated that MPOBA might inhibit EMT in CMC cells by increasing *E*-cadherin expression and decreasing *N*-cadherin and *SNAIL* expression. However, EMT-related protein expression analysis is needed to clarify the molecular mechanisms of MPOBA via the suppression of the EMT signaling pathway underlying the anticancer activity in CMCs. In addition, the mRNA expression levels of *EGFR* in both CMC cell lines were also investigated. Figure 8G and Figure 9G demonstrated that the mRNA expression level of *EGFR* significantly decreased in CMC cells treated with MPOBA compared to the control.

### 3.6. Effect of MPOBA on Expression of Vimentin (VT) and Cytokeratin (CK) in CMC Cell Lines Based on IHC Staining 

**Figure 10:**
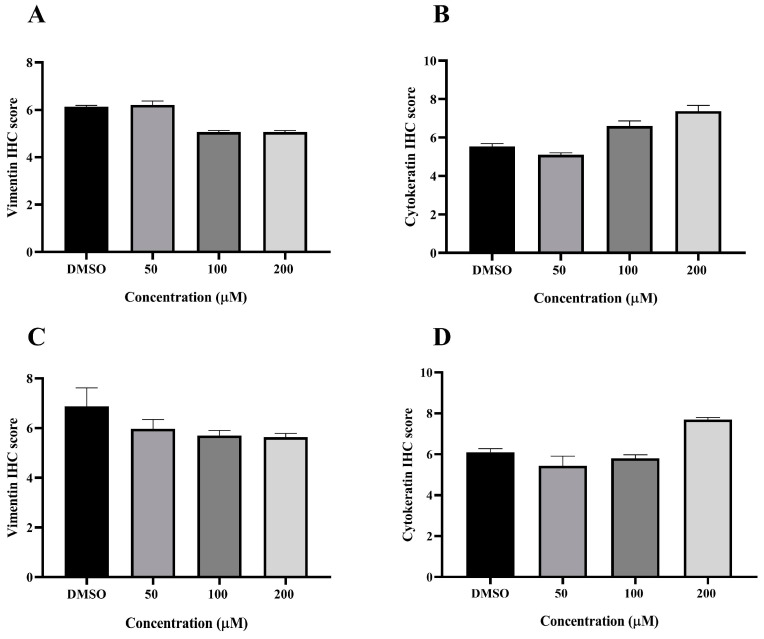
Allred scoring results of vimentin and cytokeratin immunostaining in REM134 (**A**,**B**) and CMGT071020 (**C**,**D**) cell lines.

**Figure 11:**
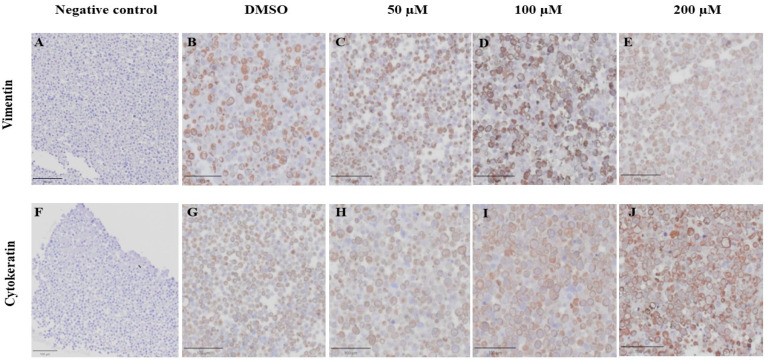
Effect of MPOBA on expression of vimentin and cytokeratin proteins in REM134 cells based on immunohistochemical staining. Negative control for vimentin (**A**) and cytokeratin (**F**) immunostaining. Vimentin immunostaining (**B**–**E**) and Cytokeratin immunostaining (**G**–**J**) in REM134 cells after treatment with MPOBA at various concentrations (40× magnification).

**Figure 12:**
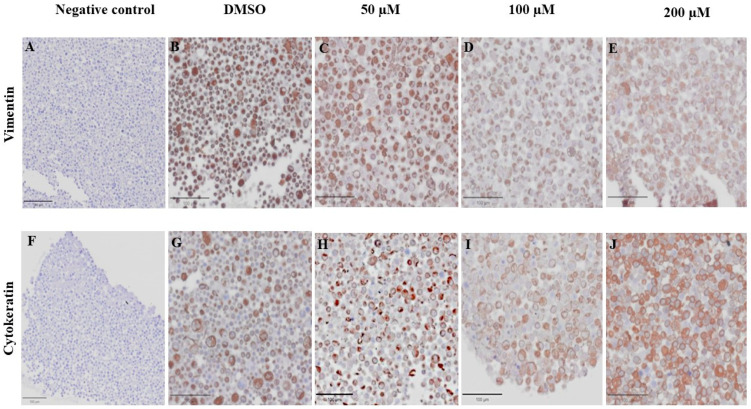
Effect of MPOBA on expression of vimentin and cytokeratin proteins in CMGT071020 cells based on immunohistochemical staining. Negative control for vimentin (**A**) and cytokeratin (**F**) immunostaining. Vimentin immunostaining (**B**–**E**) and cytokeratin immunostaining (**G**–**J**) in CMGT071020 cells after treatment with MPOBA at various concentrations (40× magnification).

VT is a major intermediate filament protein present in mesenchymal cells that is known to be overexpressed in many canine tumors and is recognized as a marker for EMT. Increased vimentin expression has been associated with tumor progression and metastasis in many cancers [49]. Meanwhile, CK is an intermediate filament protein present in epithelial cells and serves as their principal structural protein. The main function of CK is to maintain epithelial cellular architecture, and the loss of CK expression during EMT is associated with metastasis and chemoresistance [50]. The Allred score was used to evaluate VT and CK expression in CMC cells. An IHC analysis revealed that there was no statistically significant difference between the average scores of the control and treatment groups in both CMC cell lines for VT (Figure 10A,C) and CK (Figure 10B,D). Nevertheless, CMC cells treated with MPOBA exhibited a tendency toward reduced VT immunoreactivity compared to the control group (Figure 11B–F and Figure 12B–F). Likewise, the maximum CK expression was observed in CMC cells treated with MPOBA at the highest tested concentration (Figure 11G–J and Figure 12G–J). However, this was not statistically significant.

## 4. Discussion

CMC is associated with aggressive clinical behavior and high mortality in intact female dogs. Recent studies have indicated a rise in the incidence of CMC over the past few years [51]. The effectiveness of treatments for this cancer varies significantly based on the stage of cancer progression, genetic predisposition, and tolerance to chemotherapy [19]. Limitations have been concerned with the effectiveness of chemotherapy in canine patients with CMC. Consequently, there is a pressing need to develop new therapeutic agents that offer more effective treatments to enhance the outcomes of animal patients.

A variety of compounds with DO frameworks exhibit advantageous and intriguing biomedical properties, including antipsychotic, anti-inflammatory, antidepressant, insecticidal, antiepileptic, and anticancer properties [52,53]. Several of their derivatives have been documented to exhibit a promising capacity to inhibit the viability of human cancer cell lines [24]. Furthermore, it has been observed that certain derivatives of DO can induce cell cycle arrest and enhance both early and late apoptotic processes in HBC [32]. Recently, researchers became interested in an analog of DO, such as BZOP derivatives, because of their pharmacological properties. Pyridine and its derivatives play vital roles and are the most extensively applied scaffolds for drug design and synthesis [54]. Several forms of pyridine were shown, along with their biological properties, including antiviral, antimicrobial, and anticancer properties [55,56,57]. El Naggar et al. reported that pyridine-ureas showed antiproliferative activity against the MCF-7 cell line with a low IC_50_ value [58]. Kurteva also reported that a new set of amide-derivative imidazopyridine compounds exhibited anticancer properties in various human cancer cell lines, including breast cancer, lung cancer, and prostate cancer cell lines, with IC_50_ values below 1 µM [59]. Moreover, some BZOP derivatives were reported to possess anticancer activity against a human colorectal cancer cell line with an IC_50_ value of 24.95 µM [40]. Therefore, it would be worthwhile to further investigate the anticancer activities of new BZOP derivatives in CMC cell lines.

In this study, the cytotoxic effects of three BZOP derivatives, including OPDOb, OPDOc, and MPOBA, were investigated in two CMC cell lines (REM134 and CMGT071020) and a normal canine kidney cell line (MDCK) via an MTT assay. Among the tested compounds, MPOBA showed the highest antiproliferation activity against REM134 and CMGT071020 cells, with IC_50_ values of 87 and 104 µM, respectively, but low toxicity in MDCK cells. Our results indicated that MPOBA-treated cells lost their ability to proliferate in a time- and dose-dependent manner. The result suggested that MPOBA had the potential to selectively inhibit the growth of CMC cells in vitro. Therefore, this molecule was selected for the next part of the study.

Cancer metastasis consists of multi-step and multifunctional biological events that are facilitated by cell detachment, migration, invasion, and adhesion [60]. The disruption of any metastatic steps is a target for preventing the development of cancer metastases. In this study, wound healing and transwell migration assays were performed to investigate the effect of MPOBA on REM134 and CMGT071020 cell migration. Following the treatment of two CMC cell lines with MPOBA at concentrations of 100 and 200 µM for 24 h, the wound healing and transwell assays revealed reductions in the invasion and migration capabilities of both cell lines. These results indicated that MPOBA could significantly suppress the migration of both CMC cell lines in a dose-dependent manner in vitro.

Apoptosis is a fundamental biological mechanism that plays a crucial role in maintaining the balance between cell growth and cell division. One purpose of apoptosis is to eliminate uncontrolled cell proliferation, which is often associated with the development of cancer [61]. Apoptosis is characterized by a series of typical morphological changes, including cell shrinkage, chromatin condensation, and the formation of cytoplasmic blebs and apoptotic bodies [62]. In our study, MPOBA-treated CMC cells showed morphological alterations characteristic of apoptosis, including cell shrinkage, rounding, intracellular vacuolization, and a loss of cell-to-cell adhesion, over the course of 48 h. The apoptotic effect of MPOBA was examined by double staining the REM134 and CMGT071020 cells with Annexin V-FITC/PI and evaluating them using flow cytometry. The results demonstrated the significant occurrence of apoptosis in REM134 and CMGT071020 cells following the addition of MPOBA to the culture medium. Apoptosis was induced after 48 h of treatment when both CMC cell lines were treated with MPOBA at 100 µM. Based on our results, MPOBA was able to induce apoptosis in REM134 and CMGT071020 cells in a time- and dose-dependent manner. Moreover, it was also found that the percentage of early apoptosis in REM134 cells treated with MPOBA was higher than the percentages of late apoptosis and necrosis, whereas the percentage of early apoptosis in CMGT071020 cells treated with MPOBA was lower than the percentages of late apoptosis. These different findings may be dependent on the molecular subtypes of CMC cells that respond differently to MPOBA. In HBC, several studies have reported that different subtypes of HBC showed different responses to chemotherapy [63]. Thus, the classification of molecular subtypes of CMC is required for further studies to predict survival and responses to therapeutic treatments.

This study also investigated the molecular mechanism of MPOBA-induced apoptosis in both CMC cell lines by assessing the mRNA expression levels of *BAX* and *BCL-2* genes, which are widely recognized as indicators of apoptosis, and the tumor suppressor *TP53*. The *BCL-2* gene family governs the apoptosis pathway and controls mitochondrial integrity within cells [64]. The *BCL-2* gene family can be categorized into two groups based on their primary functions of (1) promoting apoptotic processes, such as *BAX*, Bcl-2 associated agonist of cell death (*BAD*), Bcl-2 homologous antagonist/killer (*BAK*), and Bcl-2 related ovarian killer (*BOK*), and (2) inhibiting apoptotic processes, such as *BCL-2*, B-cell lymphoma-extra-large (*BCL-XL*), and B-cell lymphoma-w (*BCL-W*) [65,66]. In this cellular process, *TP53* is responsible for the direct activation of the pro-apoptotic target gene *BAX*, which plays a critical role in promoting apoptosis within a cell [67]. Previous studies have demonstrated that *TP53* facilitates the transcription of *BAX* and *BAK*, which in turn regulate the release of cytochrome c from mitochondria, resulting in cell apoptosis due to the activation of caspase 3 and caspase 9 [68]. From the relative mRNA expression analysis, it was observed that the expression of the tumor suppressor *TP53* and pro-apoptotic *BAX* were upregulated, while in contrast, anti-apoptotic *BCL-2* was downregulated following MPOBA treatment. Yao et al. reported that the increased expression of BAX leads to an increase in the sensitivity of the ratio of apoptosis [69], which is similar to the results of our work. Finally, the results of our study suggested that MPOBA could sensitize these cancerous cells to apoptosis through changes in *BCL-2* and *BAX* gene expression.

Several studies have reported that many EMT-associated genes, including *SNAIL*, *SLUG*, and *TWIST*, are involved in the EMT processes of tumor invasion and metastasis [70]. *SNAIL* is a central mediator of EMT and induces EMT by directly downregulating epithelial markers such as *E-cadherin* [71]. In addition, those EMT-associated genes also increase the expression of mesenchymal phenotype markers, including *VT* and *N-cadherin*, to upregulate matrix metalloproteinases (MMPs), resulting in a tumor cell EMT and metastasis [72]. In this study, the mRNA expression levels of S*NAIL* in both MPOBA-treated CMC cell lines were significantly lower compared to the control. A significant increase in the expression level of *E-cadherin* and a decrease in the expression level of *N-cadherin* may suggest the suppression of EMT in the MPOBA-treated CMC cell lines. The inhibition of EMT by MPOBA may relate to the upregulation of *E-cadherin* and the downregulation of *N-cadherin* in CMC cells. Moreover, VT is well recognized as a hallmark of EMT, which characterizes highly invasive and metastatic carcinomas. An increase in VT expression associated with a loss of CK is an indicator of progressive cancer [73]. Based on the IHC results, there was no significant difference in IHC results between treatment and control CMC cells. However, both MPOBA-treated CMC cell lines showed a tendency towards reduced VT expression and increased CK expression compared to the control group. Therefore, additional EMT signaling-related protein expression such as N-cadherin and E-cadherin is needed to clarify the molecular mechanisms of MPOBA via the suppression of the EMT signaling pathway underlying the anticancer activity in CMCs.

In CMC cells, *EGFR* plays an important role in cellular proliferation, differentiation, and metastasis [74,75]. The inhibition of *EGFR* expression in cancer cells could inhibit the epidermal growth factor that induces increased proliferation, migration, and apoptosis [76]. An inverse correlation between the expression levels of *EGFR* and *E-cadherin* was previously demonstrated in various cancers [77]. Furthermore, the inhibition of *EGFR* expression has been reported to suppress EMT in cancer cells via the upregulation of *SNAIL* [78]. Although we observed significantly lower mRNA expression levels of *EGFR* and *SNAIL* in both CMC cell lines treated with MPOBA compared to the untreated cells, the antimigration effect of MPOBA through the *SNAIL*-mediated EMT is still unclear. Thus, further research studies should be performed to fully elucidate.

The utilization of the core structure of DO and the incorporation of additional functional groups, such as pyridine and benzaldehyde, are proposed to contribute to the specificity effect in targeting cancer cells in several studies. Numerous FDA-approved anticancer agents in humans contain a pyridine nucleus, such as Crizotinib, Regorafenib, Sorafenib, and Vismodegib [79,80]. Raslan et al. reported a novel class of 2-oxo-pyridine and 1′H-spiro-pyridine derivatives as new apoptosis-inducing agents against a colorectal adenocarcinoma (Caco-2) cell line by activation of *BAX* and deactivation of *BCL-2* genes [81]. Benzaldehyde is the simplest aromatic aldehyde molecule in various pharmaceutical compounds. In a study comparing benzaldehyde and its prodrug (5,6-benzylidene-L-ascorbate), it was found that benzaldehyde exhibited higher anticancer properties and tumor-specific cytotoxicity in human oral squamous cell carcinoma and human glioblastoma than its prodrug. The anticancer and apoptosis-inducing effects of benzaldehyde may occur through a mechanism that involves mitochondrial damage [82,83]. Additionally, a series of benzyloxybenzaldehyde derivatives, analogs of adenyl cyclase activators, showed that some of these derivatives, especially 2-[(3-methoxybenzyl)oxy]benzaldehyde, exhibited cytotoxic and anti-proliferative properties and induced apoptosis in human leukemia cells (HL-60) [84]. Recently, Tamang et al. reported that acetals of andrographolides derived from benzaldehyde enhanced their anticancer activities on HBC (MDA-MB-231 cells). These derivatives with benzaldehyde promoted apoptosis and mitochondrial damage in HBC through up-regulation of *BAX* and down-regulation of *BCL-2* expression [85]. These studies are in agreement with the findings of our work, suggesting that forming benzaldehyde groups on the main core pyridine derivatives might be considered as the important functional groups contributing to MPOBA’s activity. Its induction of apoptosis may occur through *BAX* and *BCL-2* expression and mitochondrial damage in both CMCs. However, the full elucidation of the potential anticancer effects of MPOBA, related protein expression analysis, and its target molecule in CMC still requires further investigation.

## 5. Conclusions

In summary, this study represents the first investigation into the anticancer effect of MPOBA, a new BZOP derivative, in CMC cells. Our results demonstrated that this compound significantly inhibited proliferation and migration and induced apoptosis in CMC cell lines. This effect may be regulated via a *TP53*-dependent pathway mediated by the *BAX* and *BCL-2* genes. These findings suggested that MPOBA has therapeutic potential as a mammary cancer therapy in dogs through apoptosis induction. However, additional studies are required to validate the major apoptosis signaling pathway regulated by MPOBA. In the future, we plan to continue fully elucidating the anticancer molecular mechanisms of MPOBA in CMC cells via the apoptotic pathways by analyzing the regulation of apoptosis-related protein expression and targeting the core apoptosis pathway**.** Moreover, further studies are required to comprehensively evaluate the in vivo anticancer effect of MPOBA, as well as its toxicity, prior to its further development as a potent anticancer agent.

## Figures and Tables

**Table 1:** Primer sequences used for quantitative real-time PCR (qRT-PCR).

Gene	Primer (5′ to 3′)
*SNAIL*	F: GACTCCCAGACTCGCAAGG R: GACATGCGGGAGAAGGTTCG
*SLUG*	F: GGCAAGGCGTTTTCCAGACCCT R: GGGCAAGAAAAAGGCTTCTCCCCAG
*TWIST*	F: GGCAGGGCCGGAGACCTAGATG R: TCCACGGGCCTGTCTCGCTT
*E-cadherin*	F: TCCTGGGCAGGGTGAGTT R: GAGGCCGCTTGACTGTAATC
*N-cadherin*	F: AGCACCCTCCTCAGTCAACG R: TGTCAACATGGTCCCAGCA
*TP* *53*	F: GCGGCCCAT CCTCACTATC R: CACAAACGCGTACCTCAAAGC
*EGFR*	F: CGAGCACAAGGACAACATCG R: CTCCACACATCGCTTTGGTG
*BAX*	F: GGTTGTTGCCCTCCTCTACT R: GTAAGCACTCCAGCCACAAA
*BCL-2*	F: TGGATGACTGAGTACCTGAA R: GGCCTACTGACTTCACTTAT
*GAPDH*	F: CCCACTCTTCCACCTTCGAC R: AGCCAAATTCATTGTCATACCAGG

**Table 2:** Cytotoxic effects of OPDOb, OPDOc, and MPOBA on REM134, CMGT071020, and MDCK cells after 24 and 48 h of treatment.

Compounds		Half-Maximal Inhibitory Concentration (IC_50_) (µM)				
	24 h			48 h		
	REM134	CMGT071020	MDCK	REM134	CMGT071020	MDCK
OPDOb	>300	>300	>300	172	>300	>300
OPDOc	>300	>300	>300	>300	>300	>300
MPOBA	265	>300	>300	87	104	267

## Data Availability

Data are contained within the article and Supplementary Materials.

## Supplementary Materials

The following supporting information can be downloaded at https://www.mdpi.com/article/10.3390/ani14030386/s1, Figure S1: Estimating the stain vectors in the QuPath software; Figure S2: Vimentin- and cytokeratin-positive cell detection settings and field measurements in the QuPath software [86]; Table S1: Scoring system and criteria for immunohistochemistry staining using Allred scoring method [46].
