# Supplementary material for: A New Benzo[6,7]oxepino[3,2-b] Pyridine Derivative Induces Apoptosis in Canine Mammary Cancer Cell Lines
Source: Animals (Basel). 2024 Jan 25;14(3):386. doi: 10.3390/ani14030386 (PMC10854894; doi:10.3390/ani14030386)
Supplement: Supplementary file 1 [file animals-14-00386-s001.zip › animals-2796538-supplementary.pdf]

Figure S1: Estimating the stain vectors in the QuPath software

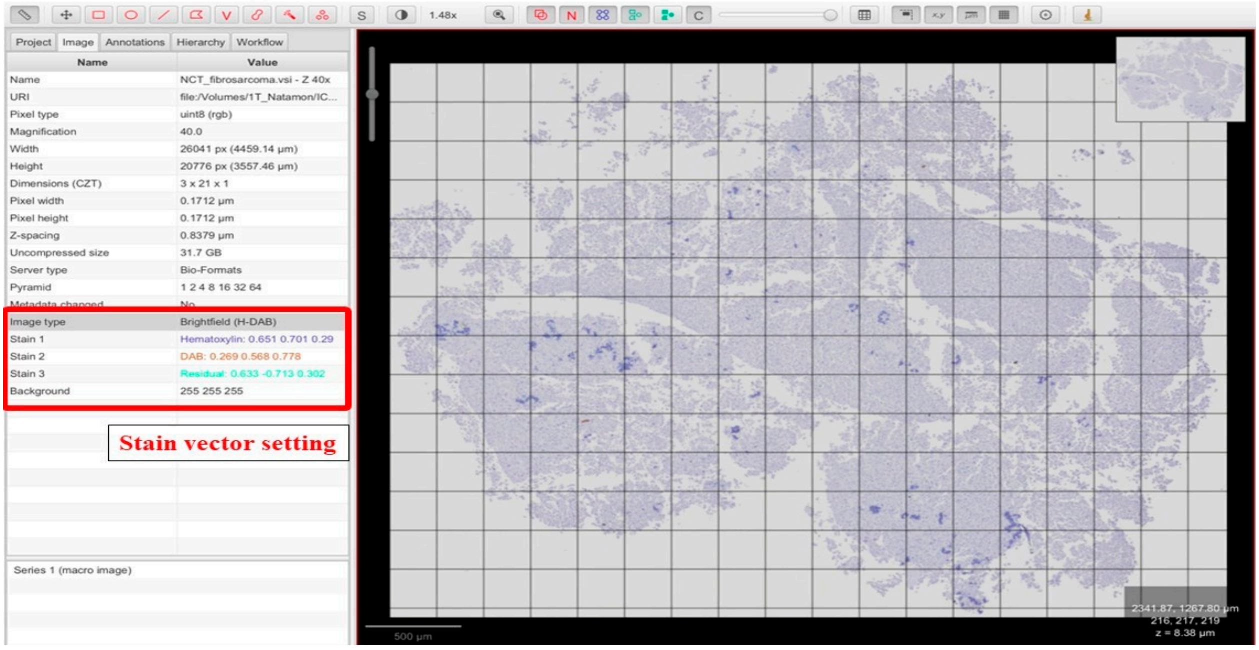

Figure S2: Vimentin- and cytokeratin-positive cell detection settings and field measurements [86] in the QuPath software

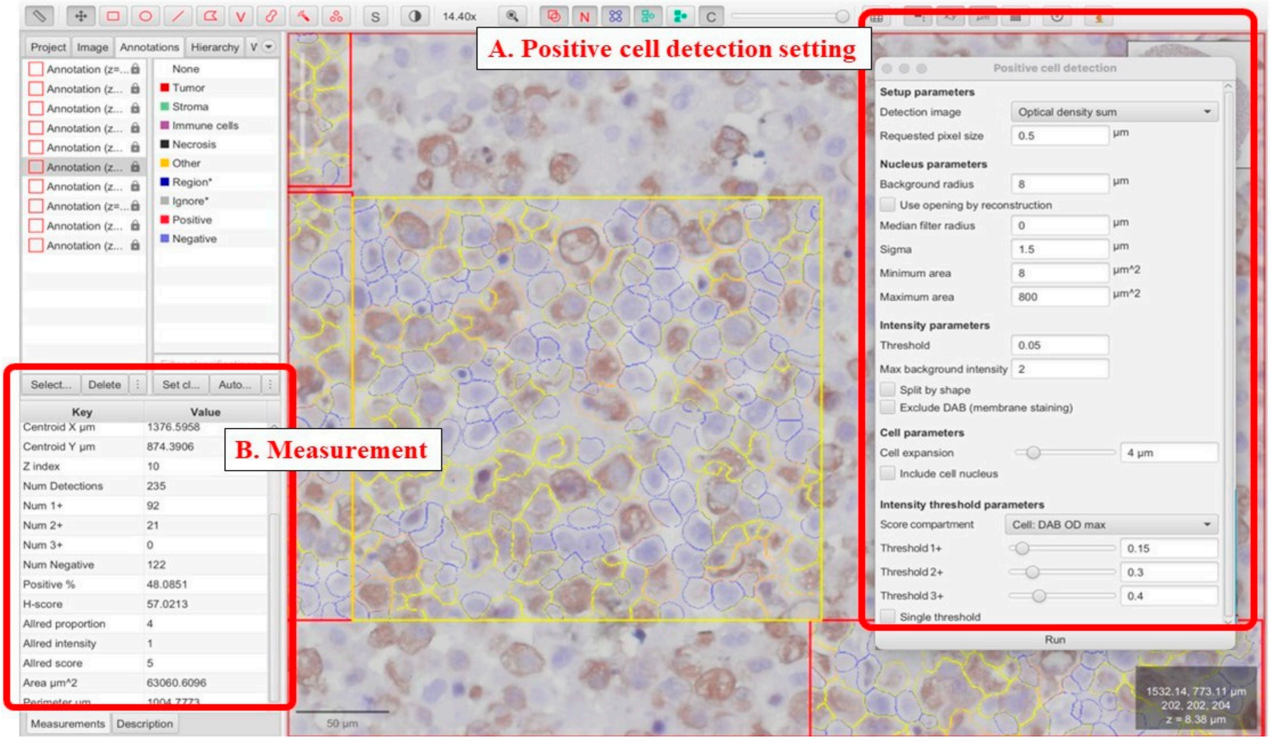

Table S1. Scoring system and criteria for immunohistochemistry staining using Allred scoring method [46].

**Proportion score (PS)**	
0	No cells are positive
1	≤ 1% of cells are positive
2	1% - 10% of cells are positive
3	11% - 33% of cells are positive
4	34% - 66% of cells are positive
5	67% - 100% of cells are positive

**Intensity score (IS)**	
0	Negative
1	Weak
2	Average
3	Strong

**Allred score interpretation (PS + IS)**	
0 – 1	Negative
2 – 3	Weak
4 – 6	Moderate
7 – 8	High
